# Durable Antibody Responses in Staff at Two Long-Term Care Facilities, during and Post SARS-CoV-2 Outbreaks

**DOI:** 10.1128/spectrum.00224-21

**Published:** 2021-07-21

**Authors:** Emily N. Gallichotte, Mary Nehring, Michael C. Young, Sierra Pugh, Nicole R. Sexton, Emily Fitzmeyer, Kendra M. Quicke, Megan Richardson, Kristy L. Pabilonia, Nicole Ehrhart, Bailey K. Fosdick, Sue VandeWoude, Gregory D. Ebel

**Affiliations:** a Department of Microbiology, Immunology and Pathology, Colorado State University, Fort Collins, Colorado, USA; b Department of Statistics, Colorado State University, Fort Collins, Colorado, USA; c Columbine Health Systems Center for Healthy Aging and Department of Clinical Sciences, Colorado State University, Fort Collins, Colorado, USA; Houston Methodist Hospital

**Keywords:** adaptive immunity, coronavirus, neutralizing antibodies, surveillance studies

## Abstract

SARS-CoV-2 has had a disproportionate impact on nonhospital health care settings, such as long-term-care facilities (LTCFs). The communal nature of these facilities, paired with the high-risk profile of residents, has resulted in thousands of infections and deaths and a high case fatality rate. To detect presymptomatic infections and identify infected workers, we performed weekly surveillance testing of staff at two LTCFs, which revealed a large outbreak at one of the sites. We collected serum from staff members throughout the study and evaluated it for binding and neutralization to measure seroprevalence, seroconversion, and type and functionality of antibodies. At the site with very few incident infections, we detected that over 40% of the staff had preexisting SARS-CoV-2 neutralizing antibodies, suggesting prior exposure. At the outbreak site, we saw rapid seroconversion following infection. Neutralizing antibody levels were stable for many weeks following infection, suggesting a durable, long-lived response. Receptor-binding domain antibodies and neutralizing antibodies were strongly correlated. The site with high seroprevalence among staff had two unique introductions of SARS-CoV-2 into the facility through seronegative infected staff during the period of study, but these did not result in workplace spread or outbreaks. Together, our results suggest that a high seroprevalence rate among staff can contribute to immunity within a workplace and protect against subsequent infection and spread within a facility.

**IMPORTANCE** Long-term care facilities (LTCFs) have been disproportionately impacted by COVID-19 due to their communal nature and high-risk profile of residents. LTCF staff have the ability to introduce SARS-CoV-2 into the facility, where it can spread, causing outbreaks. We tested staff weekly at two LTCFs and collected blood throughout the study to measure SARS-CoV-2 antibodies. One site had a large outbreak and infected individuals rapidly generated antibodies after infection. At the other site, almost half the staff already had antibodies, suggesting prior infection. The majority of these antibodies bind to the receptor-binding domain of the SARS-CoV-2 spike protein and are potently neutralizing and stable for many months. The non-outbreak site had two unique introductions of SARS-CoV-2 into the facility, but these did not result in workplace spread or outbreaks. Our results reveal that high seroprevalence among staff can contribute to immunity and protect against subsequent infection and spread within a facility.

## INTRODUCTION

The emergence of SARS-CoV-2 and the resultant COVID-19 pandemic have threatened health care systems across the world (1, 2). Long-term care facilities (LTCFs) are a significant venue for SARS-CoV-2 transmission and outbreaks, and LTCF resident deaths account for almost half of all U.S. COVID-19 deaths to date (3, 4). This is due to many factors, including the communal nature of LTCFs and the high-risk health profile of residents (5, 6). LTCF staff have the potential to introduce the virus into the facilities, where it can spread among staff, residents, and be exported back into the community. Additionally, staff at these facilities tend to resist vaccination (7–10). We therefore began weekly SARS-CoV-2 surveillance testing of staff at LTCFs and observed significant facility-associated outbreaks (11). In parallel with surveillance testing, we collected blood to determine seroprevalence, monitor seroconversion, and characterize antibody responses in these populations.

Generation of specific, neutralizing and long-lived antibodies is a key component of adaptive immunity. Studies conducted after the SARS and MERS epidemics of 2003 and 2012, respectively, revealed that the majority of recovered individuals generated antibodies; however, it is unclear whether this immunity was sufficient to provide protection against reinfection (12, 13). Many studies have sought to define the antibody response following SARS-CoV-2 infection (14, 15). These include studies on hospitalized COVID-19 patients (16–19), asymptomatic individuals (20, 21), and retrospective serological studies (22–25). The vast majority of infected individuals seroconvert and generate IgA, IgM, and IgG-specific antibodies within 3 weeks of infection (15). Age, sex, hospitalization, severity of infection, and other factors have all been shown to modulate the level, kinetics, and durability of the antibody response following infection (21, 26–32). Recent work has revealed that up to 7 months after infection, absolute binding antibody levels might decline but neutralizing antibodies appear to be long lived and persist at stable levels (33–39).

We therefore sought to characterize the antibody responses to SARS-CoV-2 in staff at two LTCFs by sampling serum at regular time intervals, both during and post outbreak. Using these samples, we measured antibody binding to two commonly used SARS-CoV-2 antigens, full-length spike and receptor-binding domain (RBD), along with neutralization of live SARS-CoV-2 virus. Our data clearly demonstrate the development of SARS-CoV-2 binding and neutralizing antibodies at approximately 1 to 2 weeks postinfection, during the period of observation for the outbreak facility. Our data also reveal that the facility with high seroprevalence did not have any outbreaks during the study period, despite the introduction of the virus into the facility on two independent occasions. These results suggest that high seroprevalence (>40%) and levels of neutralizing antibodies can contribute to outbreak resistance through community immunity. Additionally, we find that up to 4 months postinfection, neutralizing antibody levels are stable and durable.

## RESULTS

### SARS-CoV-2 surveillance testing.

**FIG 1:**
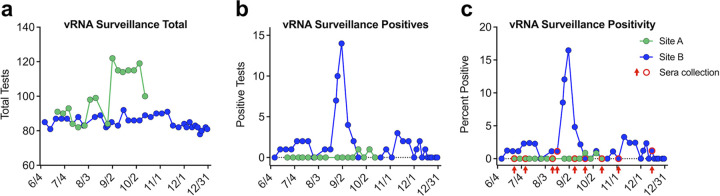
SARS-CoV-2 vRNA surveillance testing at two LTCFs. (a) Total number of staff tested weekly as part of vRNA nasal surveillance testing. (b) Number of positive vRNA tests recorded each week at sites A and B. (c) vRNA positivity expressed as percent positive at each site. Timing of sera collections relative to surveillance testing are indicated by red circles and arrows.

We performed nasal surveillance testing for SARS-CoV-2 viral RNA of staff at two long-term-care facilities over a 4 to 6 month period (Site A, July to Oct 2020; Site B, June to Dec, 2020; Fig. 1). Samples were collected at the workplace. Site A previously experienced a large outbreak in June immediately before our surveillance testing began, with 26 staff and 47 residents testing positive; however, this was before robust surveillance testing efforts were in place, so it is possible far more individuals were infected, whereas at Site B no symptomatic or asymptomatic cases had been diagnosed prior to our surveillance testing. Staff were tested at least once per week, approximately 180 unique individuals at each site participated in testing, with an average of 100 staff at site A and 85 staff at site B testing weekly (Fig. 1a). Positive tests and percent positivity varied by facility, with site A only experiencing two positive tests (from two different staff members) throughout their entire 17-week testing period (Fig. 1b and c). Site B experienced a large outbreak with over 15% of staff testing positive at its peak, and 34 unique staff testing positive throughout the 18-week study (Fig. 1b and c). We collected serum samples from staff at both sites every 3 to 5 weeks, spanning the 5-month surveillance period, including a time point immediately prior to and immediately following an outbreak in early September at site B (Fig. 1c).

### SARS-CoV-2 antibody binding and specificity.

**FIG 2:**
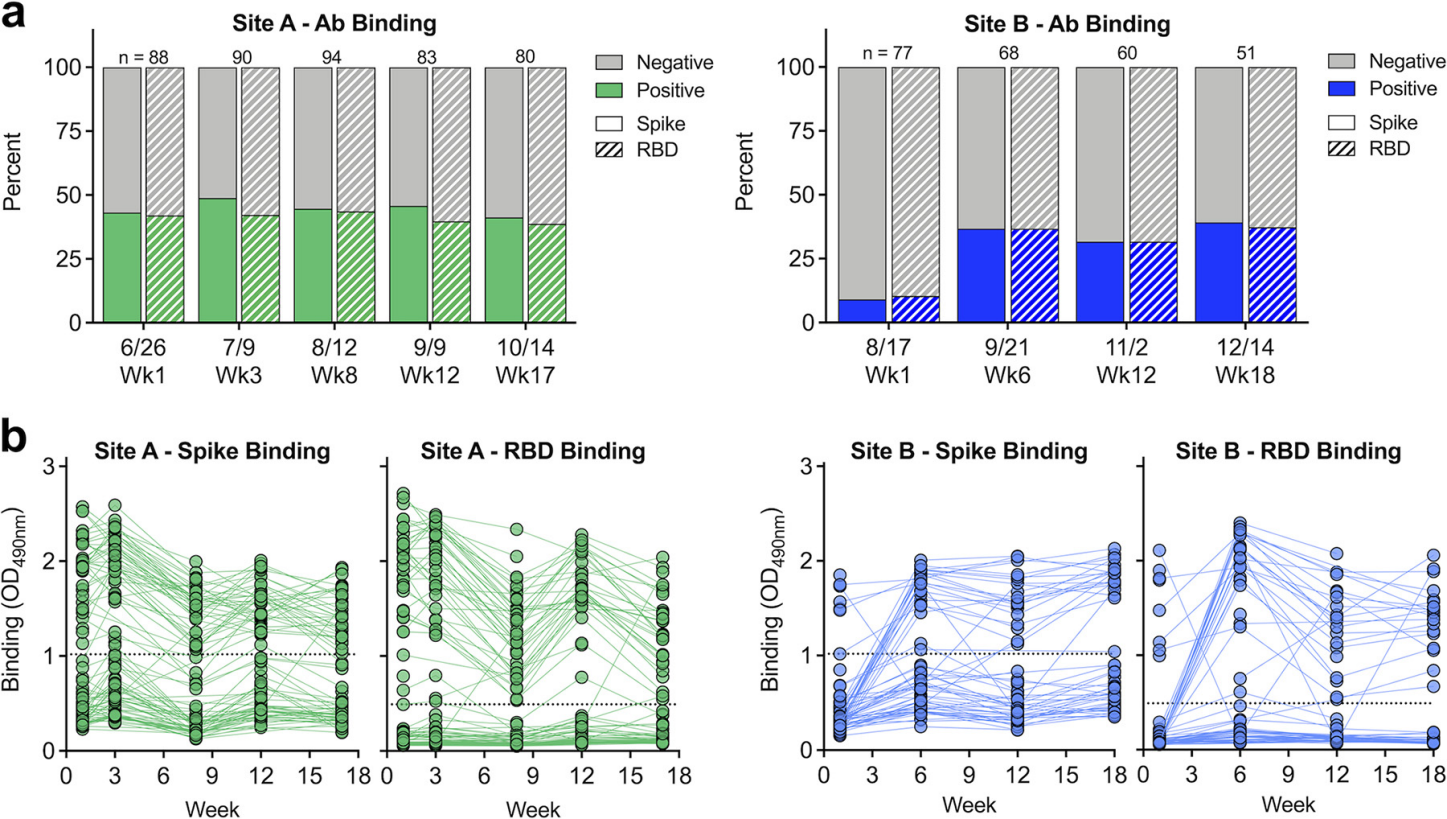
SARS-CoV-2 polyclonal antibodies bind spike and RBD. (a) Polyclonal immune sera from sites A and B were evaluated for their ability to bind recombinant spike (solid) and RBD (dash) protein; *n* indicates the number of samples tested each week. (b) Level of spike and RBD binding as determined by absorbance reading. Dashed line represents Youden cutoffs.

Sera from staff at both sites were evaluated for the presence and levels of polyclonal antibodies capable of binding to recombinant spike and receptor-binding-domain (RBD) proteins (Fig. 2a). At site A, spike and RBD binding seropositivity were approximately 40 to 50% over the 17-week study, with high agreement between the two antigens. Conversely, at site B, binding seropositivity at the start of the study, immediately prior to the large outbreak, was low (∼12%), but rapidly rose to ∼35% post outbreak (Fig. 2a). At site A, spike and RBD antibody binding levels gradually declined over the first 8 weeks, suggesting recent infection and progression from an acute to convalescent stage (Fig. 2b). At site B, binding levels quickly increased immediately following the outbreak and were stable over the following weeks (Fig. 2b).

### SARS-CoV-2 serum neutralization.

**FIG 3:**
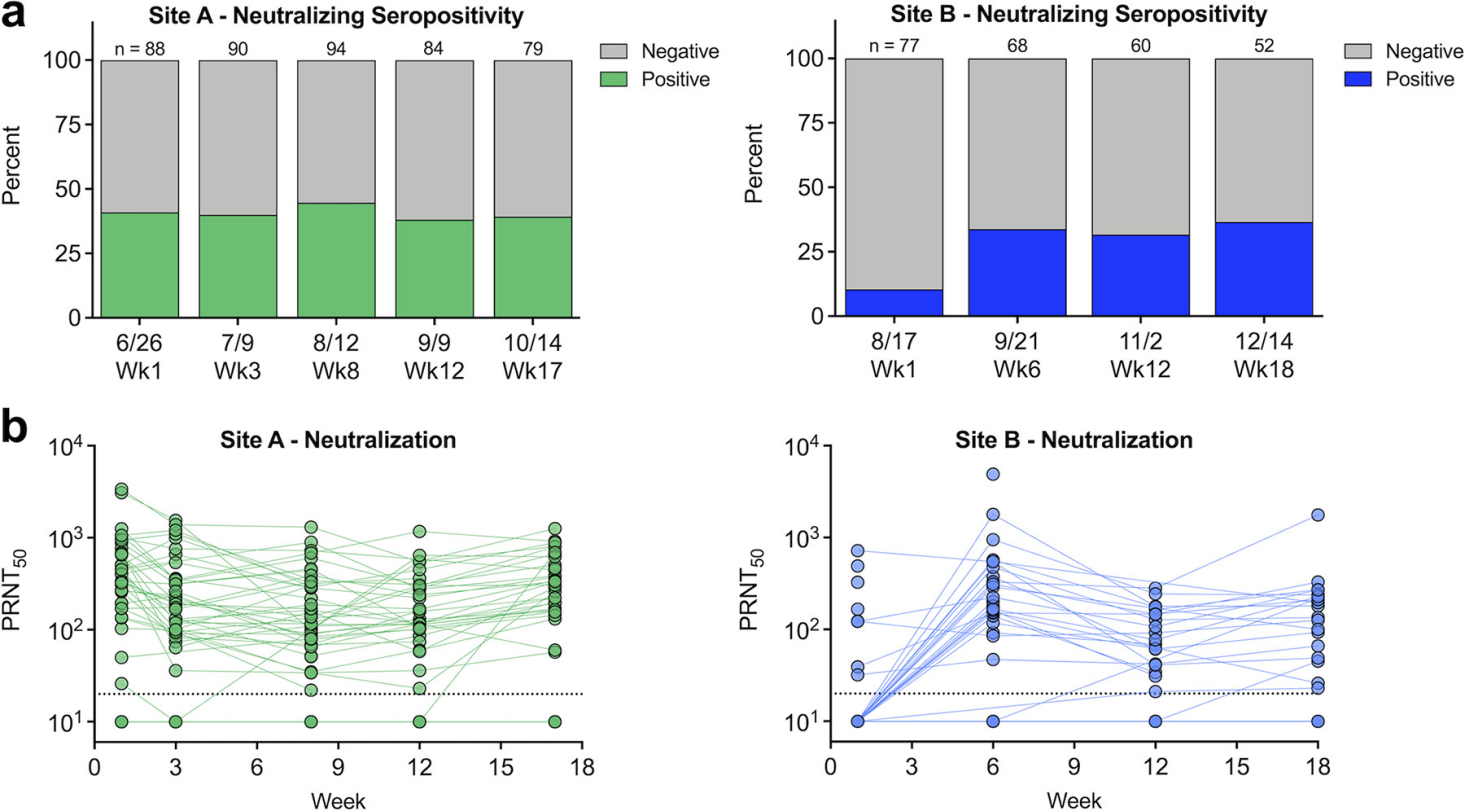
Polyclonal antibodies neutralize SARS-CoV-2 virus. (a) Polyclonal immune sera from sites A and B were evaluated for the ability to neutralize SARS-CoV-2 virus; *n* indicates the number of samples tested each week. (b) Neutralizing antibody levels over time. PRNT_50_ represents the serum dilution factor required to neutralize 50% of virus. The dashed line represents limit of detection (20). Nonneutralizing samples are graphed at half the limit of detection (10).

Sera were next evaluated for their ability to neutralize live SARS-CoV-2 virus using a standard plaque reduction neutralization test (PRNT), and their 50% neutralization titers (PRNT_50_) were calculated (Fig. 3). In agreement with antibody binding results, site A had 40 to 50% neutralizing seropositivity, which was maintained throughout the study, whereas neutralizing seropositivity at site B rapidly increased from 10% to 35% between the first sample and subsequent weeks (Fig. 3a). At site A, neutralizing titers were highly stable over the 17-week study, whereas at site B, neutralizing titers rose as individuals became infected, decreased following the acute response, and were stable during convalescence (Fig. 3b). Neutralizing antibody levels of individuals at site B who were infected prior to the beginning of the study were highly stable over the 18-week study, suggesting they were infected weeks/months prior (Fig. 3b). When analyzed using a more stringent PRNT_80_ cutoff, we saw slightly lower percent seropositivity (Fig. S1a in the supplemental material) and lower overall neutralization levels; however, the trends match the PRNT_50_ results (Fig. S1b).

### Relationship between SARS-CoV-2 polyclonal antibody binding and neutralization.

**FIG 4:**
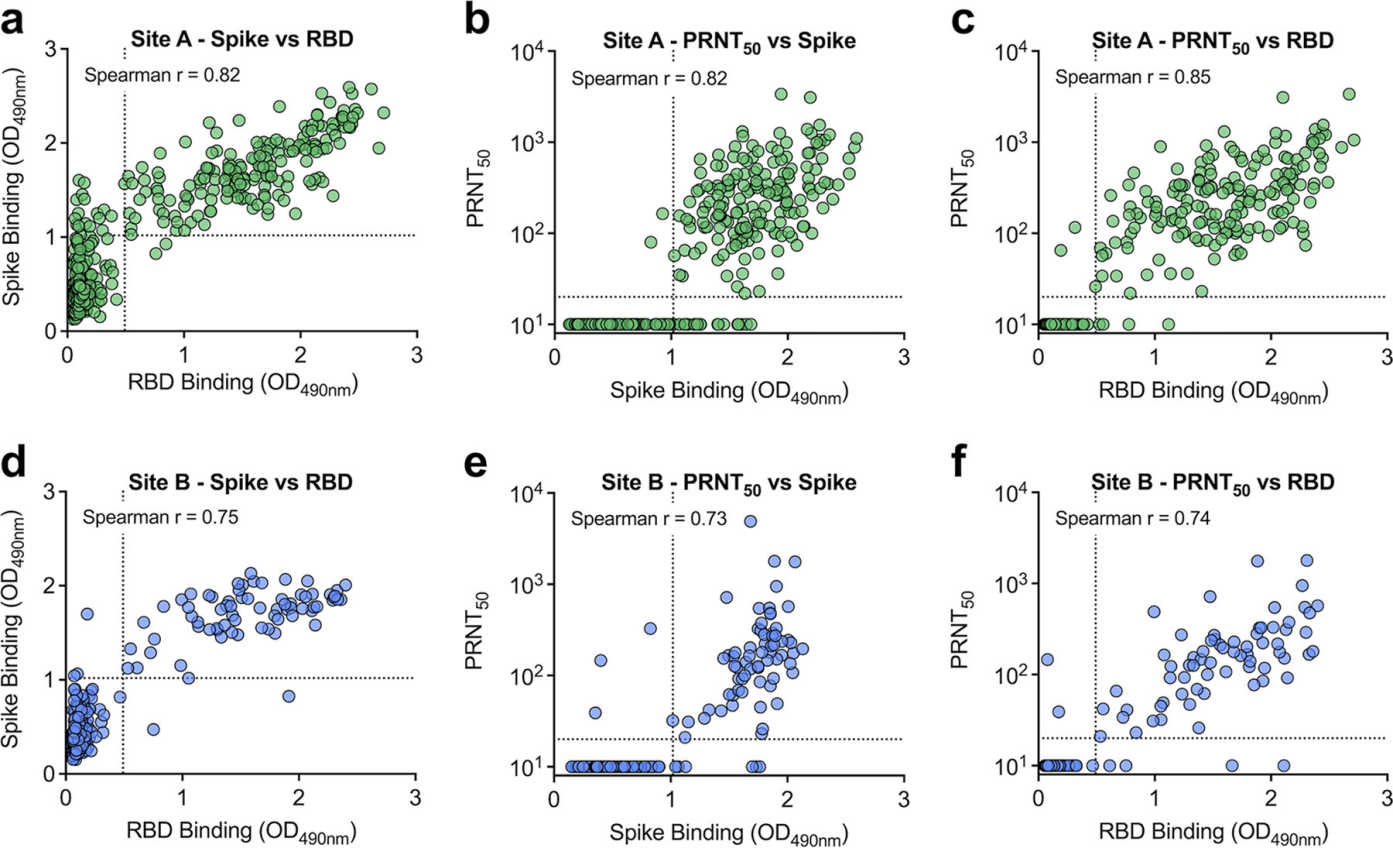
Spike binding, RBD binding, and neutralizing antibody levels are highly correlated. Samples from site A (a to c) and site B (d to f) were graphed by spike and RBD binding levels (a and d), spike binding and neutralization titers (b and e), and RBD binding and neutralization titers (c and f). Spike and RBD dashed lines represent Youden cutoffs. PRNT_50_ represents the serum dilution factor required to neutralize 50% of virus. PRNT_50_ dashed line represents limit of detection (20). Nonneutralizing samples are graphed at half the limit of detection (10). Two-tailed, nonparametic Spearman correlation is noted in the graphs.

To better understand the relationship between binding and functionally neutralizing antibodies, spike and RBD binding levels and neutralizing titers were compared (Fig. 4). At both sites, spike and RBD levels were highly positively correlated (*P* < 0.001, Spearman *r* > 0.7), suggesting the majority of spike antibodies bind within the RBD (Fig. 4a and d). At site A, there was a small population (3.9%) of samples with spike binding antibodies that were negative for RBD (Fig. 4a). Both spike and RBD antibody binding levels were highly correlated with neutralizing titers (*P* < 0.001, Spearman *r* > 0.7) (Fig. 4b, c, e, and f); however, at both sites, RBD-binding antibodies were more strongly correlated with neutralization (Fig. 4c and f). Additionally, we compared a 50% neutralization cutoff value with an 80% cutoff and found that both titers were highly correlated (Spearman *r* > 0.9) (Fig. 2a and d). When comparing the PRNT_80_ with spike and RBD binding levels, we saw strong correlations (Spearman *r* > 0.6); however, they were less strongly correlated than when using a PRNT_50_ neutralization titer (Fig. S2b, c, e, and f).

### Kinetics of SARS-CoV-2 antibody levels postinfection.

**FIG 5:**
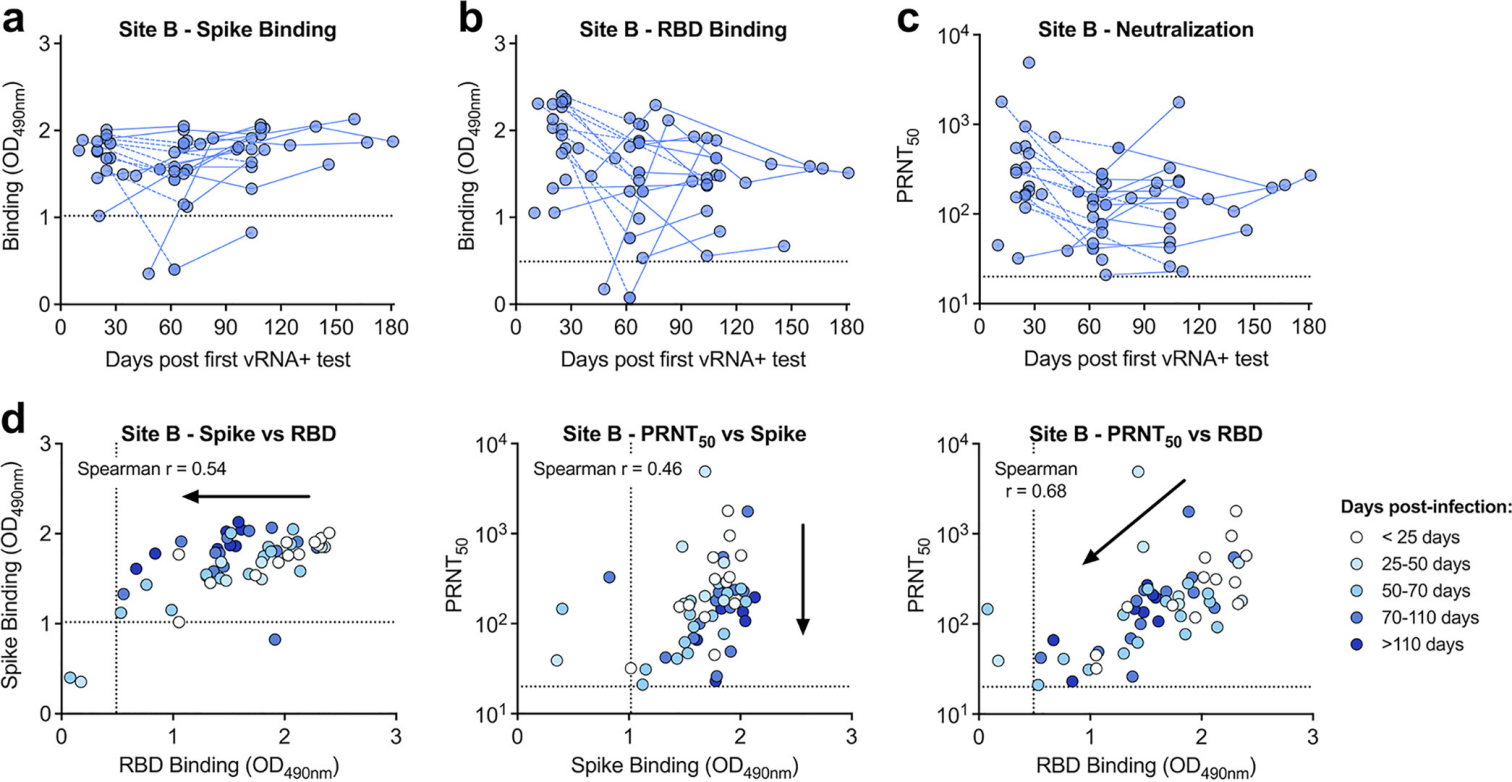
Trends in binding and neutralizing antibody levels vary over time. Individuals at site B who were infected during the course of the surveillance study were sampled up to 180 days postinfection. (a) Spike binding; (b) RBD binding; and (c) neutralizing antibody levels are graphed by days post first vRNA positive test. (d) Samples are stratified by days postinfection, and graphed by spike binding, RBD binding, and neutralization titers. Arrows show trend of data over time. Spike and RBD dashed lines represent Youden cutoffs. PRNT_50_ represents the serum dilution factor required to neutralize 50% of virus. PRNT_50_ dashed line represents limit of detection (20). Nonneutralizing samples are graphed at half the limit of detection (10). Two-tailed, nonparametic Spearman correlation is noted in the graphs.

At site B, many individuals became infected and seroconverted during the course of the study. Therefore, in these individuals, we calculated the days postinfection (first positive vRNA nasal test) relative to seroconversion and levels of antibody binding and neutralization (Fig. 5). Spike binding antibody levels were high within 30 days of a positive PCR test and remained high throughout the monitoring period (Fig. 5a). RBD binding levels were more variable and dynamic, with some individuals generating RBD-specific antibodies within 10 days following infection, whereas one individual took over 60 days to seroconvert (Fig. 5b). Neutralizing antibody titers were also variable and dynamic across individuals, though most individuals generated high levels within a month following infection (Fig. 5c, Fig. S3a). In 85% of individuals, RBD-binding and neutralizing antibody levels decreased during the first 2 to 3 months following infection and then stabilized (Fig. 5b and c, dashed lines). When comparing the relationship between binding and neutralizing antibodies stratified by timing postinfection, we again saw RBD and neutralizing antibody levels generally decrease ∼30 days postinfection, whereas spike antibodies were highly stable (Fig. 5d, Fig. S3b and c). Additionally, binding and neutralizing antibodies were highly correlated regardless of timing postinfection (*P* < 0.001).

### Phylogenetic analyses reveal lack of workplace SARS-CoV-2 spread.

**FIG 6:**
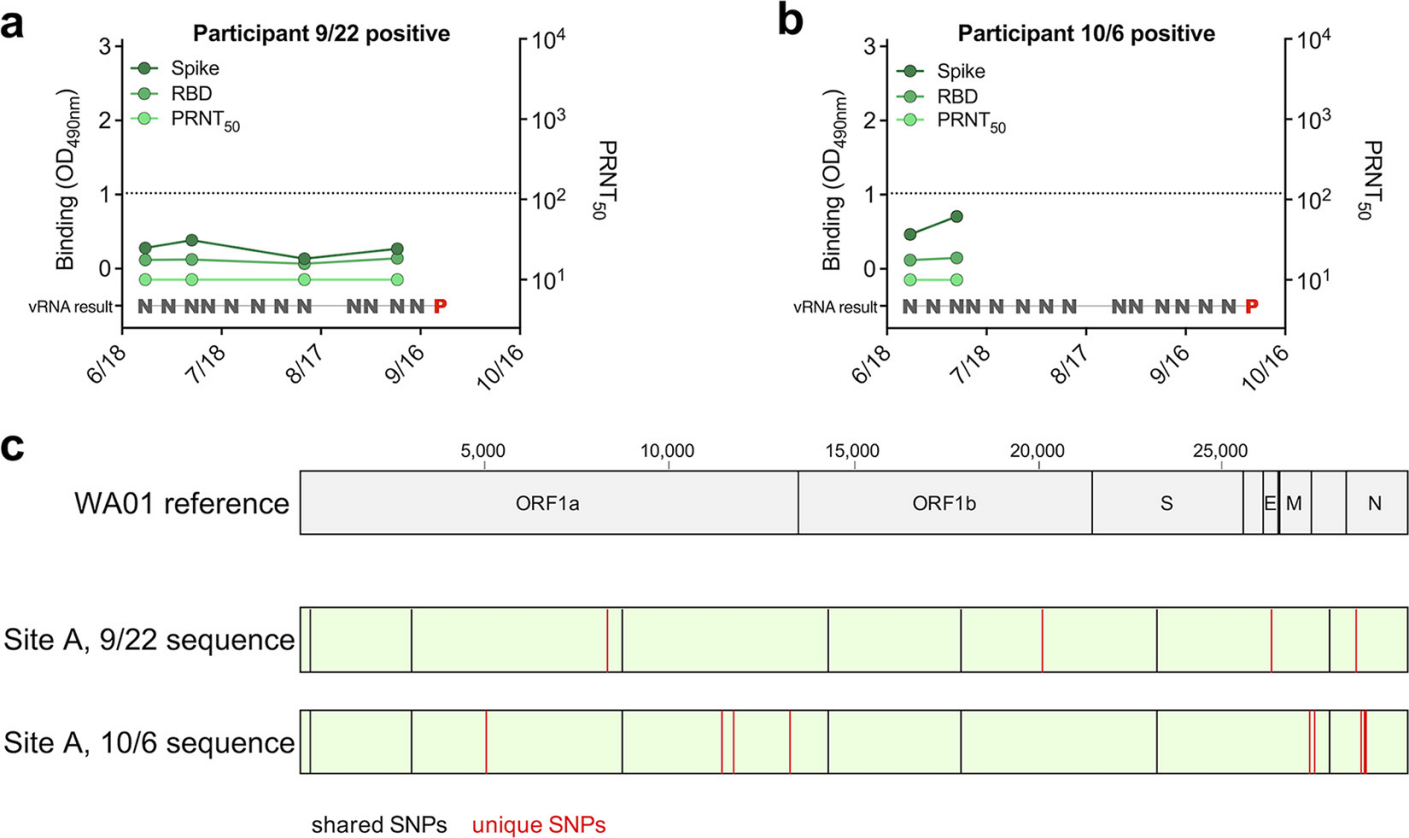
Two seronegative individuals at site A became vRNA positive with unique strains. (a and b) Spike binding, RBD binding, and neutralizing antibody levels relative to timing of surveillance vRNA testing indicated these two individuals were seronegative prior to infection (N, SARS-CoV-2 negative; P, SARS-CoV-2 positive). (c) Viral RNA from positive surveillance testing was deep sequenced and the consensus sequence was compared to the WA01 SARS-CoV-2 reference sequence. Single nucleotide polymorphisms (SNPs) shared between both site A sequences relative to the reference are shown as black lines. Unique SNPs between the site A sequences are shown as red lines.

While site A did not experience any outbreaks (infections in more than 3 individuals) during our surveillance testing, two individual staff members tested positive during our study (Fig. 1b). These infections did not result in outbreaks or spread to other staff (Fig. 2a and 3a). The two individuals who tested positive for SARS-CoV-2 vRNA (2 weeks apart on 22 September and 6 October) provided serum samples in the weeks preceding their infections. Both individuals lacked detectable binding or neutralizing antibodies prior to infection and were thus immunologically naive (Fig. 6a and b). To determine if the two viruses were genetically related, and therefore likely acquired from one another, viral genomes from the cases were sequenced. Both viruses contained shared single nucleotide polymorphisms (SNPs) relative to a reference strain (WA01); however, they also contained 13 unique SNPs that strongly distinguish one from the other (Fig. 6c), suggesting two independent infections.

## DISCUSSION

Weekly surveillance testing revealed facility-specific SARS-CoV-2 infection rates. Site B experienced a large outbreak with 34 of the staff members who participated in surveillance testing positive, whereas site A only had two positive tests out of greater than 1,600 samples total. The high infection rate of staff at site B matches the incidence rates in staff at other LTCFs during outbreaks (11, 40–42), highlighting how quickly the virus can spread among long-term care facility staff. Conversely, the low incidence of SARS-CoV-2 infection among staff at site A, paired with the high seroprevalence, suggests prior exposure and protection. Interestingly, at site A far more staff had antibodies than had previously tested positive for vRNA, suggesting a high fraction of asymptomatic infections, as has been documented in other facilities (41, 43, 44).

Two individuals at site A were vRNA positive for SARS-CoV-2 during the monitoring period. Full genome analysis of RNA recovered from these individuals revealed a significant number of genetic differences between the two isolates, suggesting they were acquired independently outside work as two unique instances of community transmission. Our finding that staff at site A had high preexisting seroprevalence (>40%) prior to intensive monitoring suggests this facility experienced a prior outbreak and had a level of immunity that limited spread of the virus from the two positive staff members (45). It is possible that other control measures and policies instituted at the time of monitoring, such as negative pressure isolation space (46), surveillance and monitoring systems, quarantine of positive staff (40, 47–49), environmental cleaning (50, 51), and other measures (52) additionally contributed to protection against outbreaks. It is notable that at both sites, seroprevalence reached a maximum of 40% during the study period, suggesting this might correspond to a level of naturally acquired immunity when coupled with other preventative measures.

Seroconversion and antibody levels were measured and characterized using three measures, i.e., binding to spike, binding to RBD, and neutralization of live SARS-CoV-2 virus. We found that immediately following infection, antibody levels peaked during the acute phase and then gradually decreased during convalescence. Neutralizing antibody levels were highly stable for at least 4 months postinfection, consistent with results reported by others (33, 37, 39). It is possible there is cross-reactivity between polyclonal antibodies from other human coronaviruses (NL63, OC43, or 229E) and SARS-CoV-2, especially when using low serum dilutions in binding assays; however, others have shown little cross-neutralization between these viruses (53–55). Antibodies that bound to spike antigen were detected earlier and more consistently than antibodies binding to RBD; however, RBD-binding antibody levels correlated most strongly with neutralizing titers, a result reported in other studies (56–60). Within our cohort, there were only four samples (0.57%) that neutralized SARS-CoV-2 but did not bind RBD. These likely neutralized through a mechanism other than blocking receptor interactions (61–63). Since RBD-binding antibodies can be detected using high-throughput platforms such as enzyme-linked immunosorbent assays (ELISAs), whereas live-virus neutralization assays require BSL3 facilities, are lower throughput, and take longer, our observations that RBD-binding antibodies are strongly correlated with neutralization suggest the more convenient binding assay may, in some circumstances, serve as a substitute for functional antiviral assays (64–68).

SARS-CoV-2 infection results in an immune response that includes the development of neutralizing antibodies (14, 15). These antibodies provide some degree of protection against reinfection with SARS-CoV-2; however, their persistence and durability are unknown, and human correlates of antibody-based protection are lacking (69–71). SARS-CoV-2 outbreaks at LTCFs can lead to high levels of seroprevalence that can limit spread within facilities (72). Without complete herd immunity, there are still nonimmune naive individuals who can become infected and spread the virus, possibly leading to secondary outbreaks (73). In our study, we observed that 40% seroprevalence in one facility, coupled with enhanced environmental controls, afforded apparent protection against subsequent outbreaks compared to a facility with low levels of preexisting seroconverted workers. Due to the high risk of infection of vulnerable individuals, staff and residents in LTCFs were among the highest priority for vaccination (74). Even in settings like LTCFs that have moderate levels of immunity acquired from natural infection, it is critical that individuals, including those previously infected, get vaccinated to further increase levels of immunity. This immunity is already drastically reducing the burden of SARS-CoV-2 in many LTCFs (75–77). Immunity, paired with additional infection control measures, will continue to reduce the incidence and prevalence of SARS-CoV-2 infection and mortality in these vulnerable facilities.

## MATERIALS AND METHODS

### Human specimens.

This study was reviewed and approved by the Colorado State University IRB under protocol number 20-10057H. Participants were consented and enrolled in our study and promptly informed of all test results. Staff represented all job classifications, including those in direct patient care roles (nurses, physical therapists, etc.) and nondirect patient care roles (custodial, administrative, etc.).

### SARS-CoV-2 vRNA surveillance testing.

Nasal swabs were collected, processed, and tested for viral RNA as described previously (11). Briefly, swabs were collected by trained personnel and placed in tubes containing viral transport medium. RNA was extracted and quantitative reverse transcriptase PCR (qRT-PCR) was performed using the CDC 2019-nCoV primers and probes (78) or the Thermo Fisher Scientific TaqPath COVID-19 combo kit, under the U.S. Food and Drug Administration (FDA) Emergency Use Authorization (EUA).

### Serum collection and processing.

Whole blood was collected in BD Vacutainer blood collection tubes (catalog number 368660). Samples were incubated for 30 to 60 min at room temperature to ensure clot formation, spun at 1,300 × *g* for 10 min at 25°C with gradual acceleration and deceleration, after which sera were aliquoted and stored at –20°C. Prior to use, sera were heat inactivated at 56°C for 30 min and then stored at 4°C.

### Spike and RBD binding assays.

RBD and spike ELISAs were modified from Amanat et al. (79). Clear, flat-bottom immune 96-well plates were coated at 2 μg/ml with SARS-CoV-2 protein (Sino) and incubated overnight at 4°C. Samples were diluted 1:50 in diluent (1% milk powder, Tween, phosphate-buffered saline [PBS]) and added to plates for 2 h at room temperature after 1 h of blocking (PBS, milk powder, Tween). Positive controls included convalescent COVID-19 patient serum (gift of Raymond Goodrich) and monoclonal antibody CR3022 (Absolute Antibody). Charcoal-inactivated pooled human serum collected in 2015 was used as a negative control (Jackson Immuno Research). Plates were washed 3× and then anti-human IgG-horseradish peroxidase (HRP) (reacts with the heavy chains of human IgG and with light chains common to most human immunoglobulins) diluted 1:3,000 (PBS, 1% milk, Tween) was added for 1 h. Plates were washed 3× and then indicator was added and incubated for 10 min (SigmaFast OPD, Sigma). Reactions were stopped with 3 M HCl and plates were read at 490 nm with a Multiskan Spectrum spectrophotometer. Raw absorbance values were not corrected or normalized to any controls.

The cutoffs for classifying ELISA results as positive/negative were based on the average optical density (OD) values across two replicates. For each binding assay, the OD cutoff was specified as that which maximizes concordance with the SARS-CoV-2 neutralization assay results, specifically that maximizing the sum of the percent positive agreement (PPA) and the percent negative agreement (PNA), akin to Youden’s index. The resulting empirical PPA and PNA were 98% and 97% for the RBD binding assay, respectively, and 99% and 92% for the spike binding assay, respectively.

### SARS-CoV-2 neutralization assay.

Vero cells were plated 1 day prior to infection. Heat-inactivated sera were serially diluted in Dulbecco’s modified Eagle medium (DMEM) containing 1% fetal bovine serum (FBS) mixed with ∼50 PFU SARS-CoV-2 (2019-nCoV/USA-WA1/2020 strain), and incubated for 1 h at 37°C. Virus-antibody mixture was added to cells, incubated for 1 h at 37°C, then overlaid with tragacanth medium. Cells were incubated for 2 days at 37°C, then fixed and stained with 30% ethanol and 0.1% crystal violet. Plaques were counted manually. Negatives controls included charcoal-inactivated pooled human serum collected in 2015 (Jackson Immuno Research) and dilution medium with no sera added. Immune sera from SARS-CoV-2 experimentally infected cats were used as positive controls (kindly gifted by Angela Bosco-Lauth).

### SARS-CoV-2 whole-genome sequencing.

Sequencing was performed as previously described (11). Briefly, cDNA was generated using SuperScript IV, PCR amplification was performed with ARTIC tiled primers and Q5 High-Fidelity polymerase. PCR products were purified and libraries were prepared using KAPA HyperPrep kit and unique index primers. Libraries were sequenced on the Illumina MiSeq V2 using 2 × 250 paired-end reads. Sequencing data were processed, quality checked, and consensus sequences were determined.
