# Supplementary material for: Durable Antibody Responses in Staff at Two Long-Term Care Facilities, during and Post SARS-CoV-2 Outbreaks
Source: Microbiol Spectr. 2021 Jul 21;9(1):10.1128/spectrum.00224-21. doi: 10.1128/spectrum.00224-21 (PMC8552744; doi:10.1128/spectrum.00224-21)
Supplement: SUPPLEMENTAL FILE 1 — Supplemental material. Download SPECTRUM00224-21_Supp_1_seq10.pdf, PDF file, 0.6 MB [file spectrum00224-21_supp_1_seq10.pdf]

Supplemental Figures

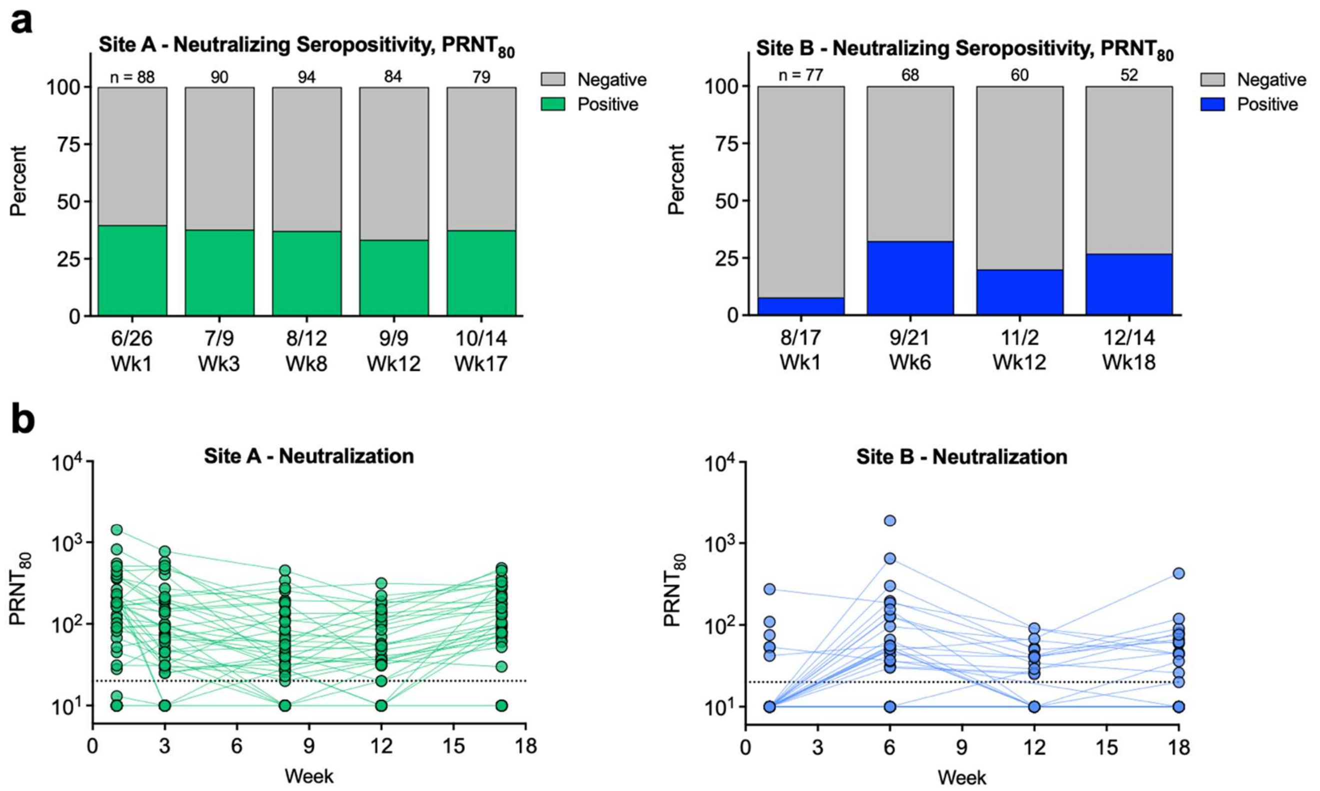

Supplemental Figure 1. Polyclonal antibodies neutralize SARS-CoV-2 virus. Polyclonal immune sera from sites A and B were evaluated for: **a)** Ability to neutralize SARS-CoV-2 virus. N indicates number of samples tested each week. **b)** Neutralizing antibody levels over time. $PRNT_{80}$ represents the serum dilution factor required to neutralize 80% of virus. Dashed line represents limit of detection (20). Non-neutralizing samples are graphed at half the limit of detection (10).

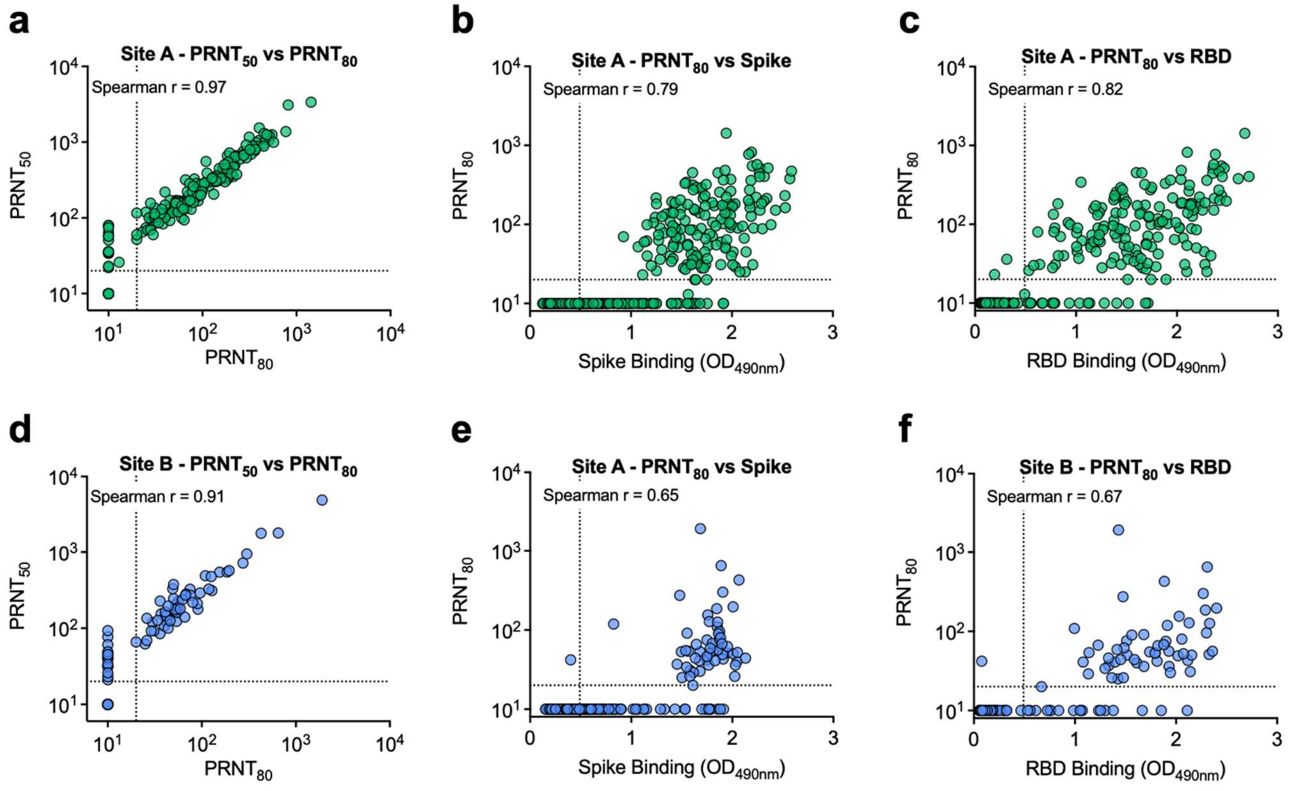

Supplemental Figure 2. Spike binding, RBD binding and neutralizing antibody levels are highly correlated. Samples from site A (**a-c**) and site B (**d-f**) were graphed by $PRNT_{50}$ and $PRNT_{80}$ (**a, d**), spike binding and neutralization titers (**b, e**) and RBD binding and neutralization titers (**c, f**). Spike and RBD dashed lines represent Youden cut-offs. $PRNT_{50}$ and $PRNT_{80}$ represents the serum dilution factor required to neutralize 50% and 80% of virus, respectively. $PRNT_{50}$ and $PRNT_{80}$ dashed line represents limit of detection (20). Non-neutralizing samples are graphed at half the limit of detection (10). Two-tailed, nonparametic Spearman correlation is noted in graphs.

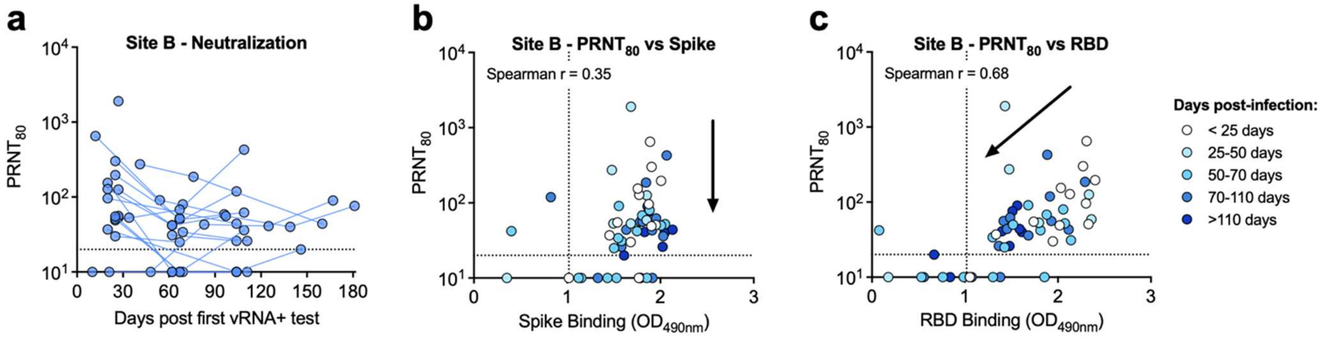

Supplemental Figure 3. Trends in binding and neutralizing antibody levels vary over time. Individuals at site B who were infected during the course of the surveillance study were sampled up to 180 days post infection. **a**) Neutralizing antibody levels are graphed by days post-first vRNA positive test. **b & c)** Samples are stratified by days post-infection, and graphed by spike binding, RBD binding and neutralization titers. Arrows show trend of data over time. Spike and RBD dashed lines represent Youden cut-offs. $PRNT_{80}$ represents the serum dilution factor required to neutralize 80% of virus. $PRNT_{80}$ dashed line represents limit of detection (20). Non-neutralizing samples are graphed at half the limit of detection (10). Two-tailed, nonparametic Spearman correlation is noted in graphs
